# Clamping of chest drain before removal in spontaneous pneumothorax

**DOI:** 10.1186/s13019-021-01398-x

**Published:** 2021-03-17

**Authors:** Yu-Hong Chan, Ellen Lok-Man Yu, Hau-Chung Kwok, Yiu-Cheong Yeung, Wai-Cho Yu

**Affiliations:** 1 Department of Medicine and Geriatrics, Princess Margaret Hospital, nil, Hong Kong, China; 2 Clinical Research Centre, Princess Margaret Hospital, Hong Kong, China

**Keywords:** Spontaneous pneumothorax, Clamping, Chest drain, Pneumothorax recurrence, Tension pneumothorax

## Abstract

**Background:**

In spontaneous pneumothorax, clamping the chest drain before its removal may avoid reinsertion in case of early recurrence, but may be unsafe and may prolong hospital stay. The objective of this study was to examine the incidence of early recurrence in both clamped and unclamped pneumothorax episodes, and factors associated with it.

**Methods:**

Retrospective chart review of primary and secondary spontaneous pneumothorax episodes in which chest drain was inserted during the period April 2012 to March 2014.

**Results:**

Data of 122 episodes were analysed. There were 36 primary pneumothorax and 86 secondary pneumothorax episodes. Mean age was 59 years with 92% males. Clamping of the chest drain was done in 68 episodes (55.7%), and not done in 54. The clamping group was significantly younger, had more primary pneumothorax, and had shorter time from cessation of air leak to clamp/removal. Recurrence within 24 h were seen in 12 (17.6%) clamped episodes and 4 (7.4%) non-clamped episodes, although in only eight episodes were reinsertion of chest drain saved. Significantly more previous pneumothorax episodes were seen in the early recurrence group. We observed no new onset of tension pneumothorax or subcutaneous emphysema associated with clamping.

**Conclusion:**

The practice of clamping the chest drain before removal in spontaneous pneumothorax appear safe. Clamping saved chest drain reinsertion in 11.8% of cases, and has the potential to save more if clamped for up to 24 h. However, clamping may result in more early recurrences. Prospective randomised studies are needed.

## Background

Pneumothorax is a clinical condition in which air is present in the pleural cavity, and was first described in detail in the nineteenth century [1]. Pneumothorax can follow trauma (traumatic pneumothorax), medical procedures (iatrogenic pneumothorax), or occur spontaneously (spontaneous pneumothorax). Spontaneous pneumothoraces is further sub-divided into primary spontaneous pneumothorax and secondary spontaneous pneumothorax based on whether there is identifiable underlying lung disease at the time that pneumothorax occurs.

For spontaneous pneumothorax, chest drain insertion is indicated for tension pneumothorax, bilateral pneumothorax, those with breathlessness, and those with failed needle aspiration [2]. Removal of the chest tube is indicated when the lung is fully expanded with no evidence of ongoing air leak. Some clinicians would remove the chest drain right away, and if pneumothorax recurs then reinsertion of chest drain is done. Others prefer to clamp the chest drain and observe for a certain period of time. The rationale for this approach is that if pneumothorax recurs the chest drain can simply be unclamped. However, there is worry that clamping may result in tension pneumothorax or subcutaneous emphysema [3, 4], and this practice may prolong hospital stay [5].

The 2003 British Thoracic Society guidelines advise that “A chest tube which is not bubbling should not usually be clamped”, adding that such practice is acceptable under the supervision of nursing staff who are trained in the management of chest drains and who have instructions to unclamp the chest drain in the event of any clinical deterioration [6]. The 2010 British Thoracic Society guidelines simply omitted to mention clamping practice [2]. On the other hand, the Belgian guidelines in 2005 recommended that “In case of doubt, a few hours of clamping with Chest X-ray (CXR) control is indicated” (by panel consensus judgement) [7]. A survey conducted by the American College of Chest Physicians showed that 47% of clinicians perform clamping for primary spontaneous pneumothorax and 59% perform clamping for secondary pneumothorax, with marked variation in practices among pulmonologists and surgeons [8]. A local multi-centre retrospective study revealed that clamping was done in 43% of pneumothorax episodes [9]. The diverse practice reflects the lack of good scientific data to provide guidance for the clinician. We have therefore performed a study to examine the efficacy and safety of clamping the chest drain before removal to provide some preliminary data for design of more definitive clinical trials.

## Methods

This was a retrospective study conducted in a single acute hospital in Hong Kong. A list of discharge episodes with patient age 18 or older, with any diagnosis of “pneumothorax” (ICD 9: 512.x), and with discharge date between 1st April 2012 to 31st March 2014 was obtained. Chart review was performed for each episode. Exclusion criteria were iatrogenic or traumatic aetiology, chest drain not inserted, patient discharged with the chest drain in-situ (transferred out, sent home, death), significant concomitant pleural effusion which affected management of the chest drain, trapped lung, incorrect coding (patient did not have pneumothorax), and insufficient data.

For eligible patients, relevant demographic and clinical data were collected. Estimation of size of pneumothorax was according to Collins’s method [10]. The CXR prior to clamping or removal of the CD was reviewed to ensure that the lung had in fact fully expanded. Whether or not clamping was done was recorded. The primary outcome was recurrence of pneumothorax within 24 h with or without clamping. Secondary outcomes were proportion of chest drain reinsertion saved by clamping, and incidence of events thought to be possibly related to clamping, including new onset of tension pneumothorax and subcutaneous emphysema.

The incidence of pneumothorax recurrence within 24 h with 95% confidence interval was calculated. Independent t-test, Mann-Whitney U test, Pearson’s chi-square test or Fisher’s exact test were used to compare subjects’ socio-demographic and clinical factors between clamping group and non-clamping group, as well as between early recurrence and late or no recurrence cases. Statistical analyses were performed using SPSS 22.0 for Windows (IBM Corp., Armonk, New York) and statistical significance was set at *p* < 0.05.

The study was approved by the Kowloon West Cluster Research Ethics Committee of Hong Kong Hospital Authority.

## Results

**Fig. 1:**
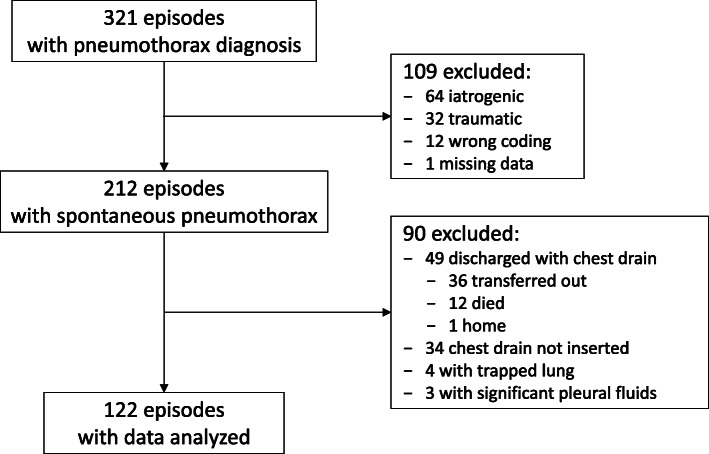
Subject recruitment flow chart

Between April 2012 and March 2014, there were 321 discharge episodes with diagnosis of “pneumothorax”. Among them, 109 episodes were excluded because of non-spontaneous pneumothorax diagnosis, incorrect coding, or with missing data. Of the remaining 212 episodes of spontaneous pneumothorax, 90 were excluded, mainly because the patient was transferred out with the chest tube, or chest tube was not inserted. Data of the remaining 122 episodes were analysed. Details are shown in Fig. 1.

The mean age of the cohort was 59.2 years. The majority were males (91.8%) and had secondary pneumothorax (70.5%). Of the 86 secondary pneumothorax episodes, 70 (81.4%) had chronic obstructive pulmonary disease with or without other lung conditions. Pneumothorax was loculated in three of 36 primary pneumothorax episodes and 70 of 86 secondary pneumothorax episodes. For the 49 episodes of non-loculated pneumothoraces, mean size immediately before chest tube insertion was 56.6% of the hemithorax volume (range 14.5–100%). Other details are shown in Table 1.

**Table 1:** Baseline characteristics of subjects with and without clamping of chest tube before removal

	All		Clamping of chest tube				*P*-value*
			No		Yes		
	(n = 122)		(*n* = 54)		(*n* = 68)		
Age (year)	59.2 ± 22.9		64.8 ± 20.1		54.8 ± 24.1		0.014
Male sex	112	(91.8)	49	(90.7)	63	(92.6)	0.749
Smoking^a^							0.284
Never	20	(16.5)	6	(11.3)	14	(20.6)	
Ex-smoker	69	(57.0)	34	(64.2)	35	(51.5)	
Current smoker	32	(26.4)	13	(24.5)	19	(27.9)	
Charlson comorbidity index	1.8 ± 2.0		1.7 ± 2.0		1.9 ± 2.1		0.484
Secondary pneumothorax	86	(70.5)	46	(85.2)	40	(58.8)	0.002
Side of pneumothorax							0.570
Right	47	(38.5)	23	(42.6)	24	(35.3)	
Left	74	(60.7)	31	(57.4)	43	(63.2)	
Bilateral	1	(0.8)	0		1	(1.5)	
Size of pneumothorax^b^	56.6 ± 24.9		53.0 ± 26.0		59.1 ± 24.2		0.404
No. of previous episodes							0.229
0	75	(61.5)	35	(64.8)	40	(58.8)	
1	29	(23.8)	15	(27.8)	14	(20.6)	
2	10	(8.2)	2	(3.7)	8	(11.8)	
≥ 3	8	(6.6)	2	(3.7)	6	(8.8)	
Total no. of chest tube(s)							0.211
1	106	(86.9)	44	(81.5)	62	(91.2)	
2	11	(9.0)	6	(11.1)	5	(7.4)	
≥ 3	5	(4.1)	4	(7.4)	1	(1.5)	
Prior medical pleurodesis	61	(50)	29	(53.7)	32	(47.1)	0.466
Type of chest tube							0.456
≥ fr 18	70	(57.4)	29	(53.7)	41	(60.3)	
fr 12	51	(41.8)	24	(44.4)	27	(39.7)	
fr 8	1	(0.8)	1	(1.9)	0		
Clinical setting							0.006
Medical	116	(95.1)	48	(88.9)	68	(100)	
Non-medical	6	(4.9)	6	(11.1)	0		
Duration of cessation of air leak before clamping/ removal (hour)^d^	45.3 ± 33.6		58.3 ± 37.5		35.3 ± 26.5		< 0.001
Duration of drainage (day)	10.5 ± 13.5		12.2 ± 15.7		9.1 ± 11.3		0.228

Data are presented as mean ± SD or count (%)

* Independent t-test, Pearson’s chi-square test or Fisher’s exact test

^a^ n (non-clamping) = 53

^b^ For non-loculated pneumothorax only: n (non-clamping) = 20, n (clamping) = 29

^d^ n (non-clamping) = 52, n (clamping) = 67

In 68 episodes (55.7%) the chest drain was clamped prior to removal, while in the remainder (54, 44.3%) it was removed without clamping. By univariable analysis, the clamping group was significantly younger, had more primary pneumothorax episodes, shorter time from cessation of air leak to clamp/removal, and fewer episodes managed in non-medical ward setting. All other demographic and disease metrics were comparable. (Table 1). Multivariable analysis was not performed because of the small sample size. For those with clamping, new development of tension pneumothorax, subcutaneous emphysema, and displacement of the chest tube were not seen.

**Fig. 2:**
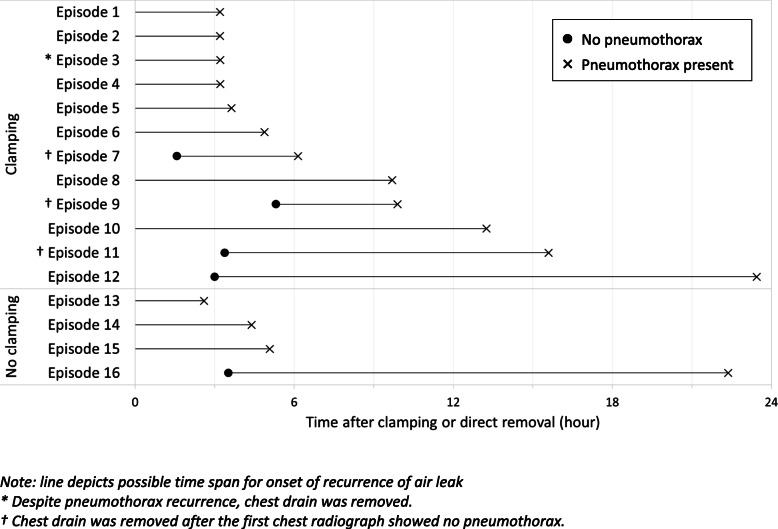
Timing of pneumothorax recurrence of the 16 episodes

Recurrence of pneumothorax within 24 h occurred in 16 episodes (13.1, 95% CI 8.2–20.2%), with 12 in the clamping group (17.4% of 68), and 4 in the non-clamping group (7.4% of 54). The difference was not statistically significant (*p* = 0.096). The time from clamping or removal of the chest drain to detection of pneumothorax recurrence for these 16 episodes were depicted in Fig. 2. Of the 12 clamped episodes, chest drain re-insertion was saved in eight episodes (11.8% of 68, 95% CI 6.1–21.5%). For the remaining four episodes, one (episode 3 in Fig. 2) had pneumothorax recurrence detected at 3.22 h, but the chest drain was nonetheless removed. In three episodes (7, 9, 11 in Fig. 2) the first CXR showed no recurrence of pneumothorax, and their chest drains were duly removed. Incidentally, episode 12 had first CXR at 3 h showing no recurrence of pneumothorax, but the chest drain was retained and clamping continued. CXR at 23.45 h showed recurrence of pneumothorax, and the chest drain was unclamped.

When comparing the demographic and clinical characteristics between those with pneumothorax recurrence within 24 h and the rest of the cohort, there was significantly more previous pneumothorax episodes in the early recurrence group. All other parameters were essentially comparable. (Table 2).

**Table 2:** Comparison between subjects with and without recurrence within 24 h (*n* = 122)

	Early recurrence (within 24 h)				*P*-value*
	No		Yes		
	(*n* = 106)		(*n* = 16)		
Age (year)	58.9 ± 22.7		61.4 ± 24.3		0.135
Male sex	98	(92.5)	14	(87.5)	0.619
Smoking^a^					1.000
Never	18	(17.1)	2	(12.5)	
Ex-smoker	59	(56.2)	10	(62.5)	
Current smoker	28	(26.7)	4	(25)	
Charlson comorbidity index	1.8 ± 2.1		1.9 ± 1.9		0.581
Secondary pneumothorax	74	(69.8)	12	(75)	0.776
Side of pneumothorax					0.644
Right	42	(39.6)	5	(31.3)	
Left	63	(59.4)	11	(68.8)	
Bilateral	1	(0.9)	0		
Size of pneumothorax^b^	57.2 ± 25.2		50.3 ± 23.5		0.104
No. of previous episodes					0.023
0	69	(65.1)	6	(37.5)	
1	21	(19.8)	8	(50)	
2	10	(9.4)	0		
≥ 3	6	(5.7)	2	(12.5)	
Total no. of chest tube(s)					0.270
1	93	(87.7)	13	(81.3)	
2	8	(7.5)	3	(18.8)	
≥ 3	5	(4.7)	0		
Prior medical pleurodesis	53	(50)	8	(50)	1.000
Type of chest tube					0.152
≥ fr 18	62	(58.5)	8	(50)	
fr 12	44	(41.5)	7	(43.8)	
fr 8	0		1	(6.3)	
Clinical setting					0.578
Medical	101	(95.3)	15	(93.8)	
Non-medical	5	(4.7)	1	(6.3)	
Duration of cessation of air leak before clamping/ removal (hour)	47.2 ± 34.4		33.2 ± 25.5		0.221
Duration of drainage (day)	10.3 ± 12.5		11.7 ± 19.2		0.596

Data are presented as mean ± SD or count (%)

* Mann-Whitney U test, Pearson’s chi-square test or Fisher’s exact test

^a^ n (no early recurrence) = 105

^b^ For non-loculated pneumothorax only: n (no early recurrence) = 45, n (early recurrence) = 4

## Discussion

With the lack of international guidelines on clamping prior to removal of chest drains in spontaneous pneumothorax, it is hardly surprising that clinical practice varies widely between different places in the world, and even within individual institutions. Clamping rate varies from “infrequent” in a London teaching hospital [11], “very occasionally” in City Hospital, Nottingham, United Kingdom [12], and 24% in Ulster Hospital, Northern Ireland [13], to more than half in studies performed in the Czech Republic [14], Wales [15], and the United States [8]. The clamping rate in this study is 56%. Thus, although some authors rejected this practice on grounds that it prolongs hospital stay and could be dangerous [3–5], physicians and surgeon around the world continue to do it without apparent problem.

In our cohort of 68 episodes with chest tube clamping, no new tension pneumothorax or subcutaneous emphysema were seen. This is in agreement with our clinical experience that such events are exceedingly uncommon. The claim that clamping of the chest drain may result in tension pneumothorax was not supported by good rationale [16]. Indeed, we have been unable to find a single case report of onset of tension pneumothorax or subcutaneous emphysema following clamping of the chest drain after detailed literature search.

Hong Kong do not have a regional guideline on clamping practice, and whether clamping is done is left to individual clinician preference. We have not done a survey to examine the rationale of their choices, and we are not aware of published data in this area. By univariable analysis, the clamping group has significantly younger patients, more primary pneumothorax cases, and shorter time from cessation of air leak to chest drain intervention. These three metrics are likely related in that patients with primary spontaneous pneumothorax are younger and has fewer medical pleurodesis performed, hence the short time from cessation of air leak to intervention to remove the chest drain. There was in fact higher proportion of pleurodesis episode in the unclamped group, though the different was not statistically significant. This observation may be explained by the perceived lower chance of early recurrence with longer time from cessation of air leak. Nevertheless, these findings need to be examined in larger studies. All six episodes managed in non-medical wards did not have clamping done, and this reflects the clinical practice of non-medical specialties in our hospital.

There were 16 pneumothorax recurrences occurring within 24 h of the decision to either remove or clamp the chest drain. By univariable analysis, these 16 cases had significantly higher proportion of recurrent pneumothorax cases, but this need to be confirmed by more data. In only eight episodes (11.8% of 68 episodes) were re-insertion of the chest drain saved, as detailed in the results section. Had all 68 episodes been clamped for up the 24 h, the remaining four episodes might have been saved from chest drain reinsertion as well, elevating the proportion to 17.6%. Are these benefits worth the cost of the inevitable increase in length of hospital stay for the over 80% of clamped cases without early recurrence? Further studies with cost-effectiveness considerations is needed.

We could find only one report in the literature which is similar to our study. It involved 243 trauma patient with pneumothorax, haemothorax, or both [17]. One hundred and thirty-four patients (55.1%) underwent clamping and 109 did not. Thirteen patients (9.7% of clamping group) had recurrence shown in the CXR taken at 6 h and required unclamping. A further nine patients (6.7%) who had passed the clamping trial required reinsertion of chest drain later. No adverse events related to clamping was observed. Although the disease entities are not the same, the main findings are very similar to our study, in particular that CXR taken at 6 h is likely to miss a significant number of early recurrence cases. Interestingly, in the study by Funk et al. only five (4.6%) who did not undergo clamping had early pneumothorax recurrence requiring reinsertion of the chest drain, which is significantly fewer than the 22 patients (16.4%) in the clamping group. This trend is also observed in our study. Whether clamping is associated with increased risk of early pneumothorax recurrence may be worthy of further exploration.

There are limitations to our study. Firstly, clamped and unclamped cases were not randomised. It follows that comparisons between these two groups, particularly the clinical outcome, should be interpreted with caution. Secondly, being a retrospective study, some data, especially the exact time of cessation of air leak, may not be entirely accurate. Thirdly, there was no standard management protocol so that data like CXR time post-intervention varied widely. Fourthly, the vast majority of patient were males hence the data may not apply to females. Fifthly, to mix up primary and secondary pneumothorax cases in the same cohort may be problematic, as these two disease entities are different in many clinical aspects. Finally, the sample size is not large enough for more stringent statistical handling of the data, so that many of the findings can only be considered preliminary.

## Conclusions

In conclusion, for spontaneous pneumothoraces, clamping of chest drains before removal appear safe. Clamping as per current clinical practice saves reinsertion of the chest drain in 11.8% of cases, and has the potential to save more if clamped for up to 24 h. However, it may be associated with increased early pneumothorax recurrence. Prospective randomised study with adequate sample size is needed to provide more data on safety, efficacy, and cost-effectiveness.

## Data Availability

The datasets generated and/or analysed during the current study are not publicly available as it is owned by the Hospital Authority but are available from the corresponding author on reasonable request.

## Abbreviations

CXR: Chest X-ray
ICD: International Classification of Diseases
CD: Chest drain
SD: Standard Deviation
